# Whitebark pine facilitation at treeline: potential interactions for disruption by an invasive pathogen

**DOI:** 10.1002/ece3.2198

**Published:** 2016-06-28

**Authors:** Diana F. Tomback, Sarah C. Blakeslee, Aaron C. Wagner, Michael B. Wunder, Lynn M. Resler, Jill C. Pyatt, Soledad Diaz

**Affiliations:** ^1^ Department of Integrative BiologyCampus Box 171University of Colorado DenverPO Box 173364DenverColorado80217; ^2^ Department of GeographyVirginia Tech115 Major Williams Hall (0115)BlacksburgVirginia24061

**Keywords:** *Cronartium ribicola*, facilitation, leeward microsites, *Pinus albicaulis*, Rocky Mountains, seedlings, shoot lengths, stress tolerance, tree islands, treeline

## Abstract

In stressful environments, facilitation often aids plant establishment, but invasive plant pathogens may potentially disrupt these interactions. In many treeline communities in the northern Rocky Mountains of the U.S. and Canada, *Pinus albicaulis*, a stress‐tolerant pine, initiates tree islands at higher frequencies than other conifers – that is, leads to leeward tree establishment more frequently. The facilitation provided by a solitary (isolated) *P. albicaulis* leading to tree island initiation may be important for different life‐history stages for leeward conifers, but it is not known which life‐history stages are influenced and protection provided. However, *P. albicaulis* mortality from the non‐native pathogen *Cronartium ribicola* potentially disrupts these facilitative interactions, reducing tree island initiation. In two Rocky Mountain eastern slope study areas, we experimentally examined fundamental plant–plant interactions which might facilitate tree island formation: the protection offered by *P. albicaulis* to leeward seed and seedling life‐history stages, and to leeward krummholz conifers. In the latter case, we simulated mortality from *C. ribicola* for windward *P. albicaulis* to determine whether loss of *P. albicaulis* from *C. ribicola* impacts leeward conifers. Relative to other common solitary conifers at treeline, solitary *P. albicaulis* had higher abundance. More seeds germinated in leeward rock microsites than in conifer or exposed microsites, but the odds of cotyledon seedling survival during the growing season were highest in *P. albicaulis* microsites. Planted seedling survival was low among all microsites examined. Simulating death of windward *P. albicaulis* by *C. ribicola* reduced shoot growth of leeward trees. Loss of *P. albicaulis* to exotic disease may limit facilitation interactions and conifer community development at treeline and potentially impede upward movement as climate warms.

## Introduction

During the last 20 years, numerous studies have demonstrated the importance of facilitation interactions to plant survival and regeneration in stressful environments (Bertness and Callaway 1994; Lortie et al. 2004; Brooker et al. 2008). Stressful conditions especially characterize high‐elevation communities. Callaway et al. (2002) examined 115 plant species in 11 mountain sites globally and found that competitive interactions at lower elevations transition to facilitative interactions between the same species at higher elevations.

Conifer seedling establishment in the Rocky Mountain alpine treeline ecotone occurs under conditions of high winds, cold and variable temperatures, short growing seasons, poorly developed soils, variable snowpack, variable water availability, and intense solar radiation (Marr 1977; Arno and Hammerly 1984; Holtmeier 2003; Smith et al. 2003; Maher et al. 2005; Körner 2012). Survival is improved when extreme climatic conditions are mitigated by windward shelter, such as rocks, topographic niches, and other “nurse objects,” or when an established conifer provides protection for conifers growing in its lee (Callaway 1998; Hättenschwiler and Smith 1999; Germino et al. 2002; Resler et al. 2005; Resler and Tomback 2008; Batllori et al. 2009).

Although we understand the importance of facilitation in climatically stressful environments, such as the temperate zone alpine treeline ecotone, we have yet to understand the consequences to community structure and composition of altered interactions through infestation by exotic pathogens and pests. With increasing globalization and warming temperatures, outbreaks of non‐native pests and diseases are impacting plant health globally, and potentially disrupting community interactions (Boyd et al. 2013; Weed et al. 2013; Roy et al. 2014).

**Figure 1:**
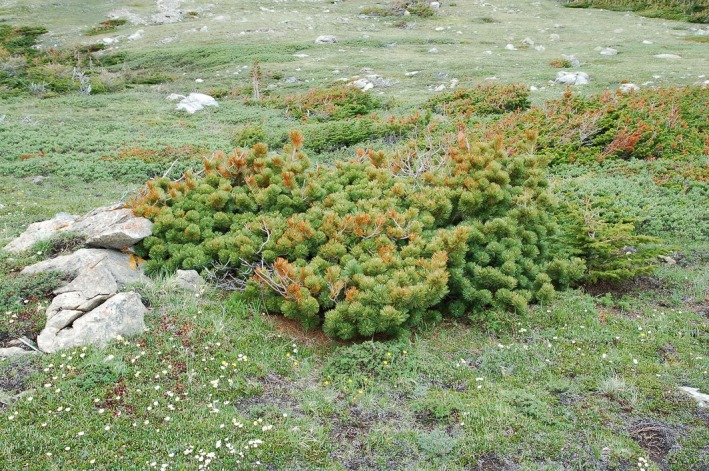
Krummholz *Pinus albicaulis* (whitebark pine) on Divide Mountain growing windward of other conifers within a tree island. Photograph credit: D. F. Tomback.

In the Rocky Mountains, *Pinus albicaulis* (whitebark pine) is a common subalpine and treeline conifer (Fig. 1). A poor competitor on productive sites, *P. albicaulis*, tolerates nutrient‐poor soils, aridity, and cold temperatures (Arno and Hoff 1990). It grows slowly, has moderately long leaf persistence, and first produces seed cones at 20–30 years of age (Krugman and Jenkinson 1974). These traits conform to the “stress‐tolerant” strategy of Grime (1977), who classified alpine habitats as one of four severely stressful conditions for plants. McCune (1988) definitively classified *P. albicaulis* as a stress‐tolerant pine, which is recently supported by specific physiological characteristics, including greater carbon gain and water use efficiency (Callaway et al. 2000; Bansal et al. 2011).

In many treeline krummholz communities, *P. albicaulis* functions as the most common tree island initiator and thus occurs as the windward conifer more frequently than other conifers (Resler and Tomback 2008; Resler et al. 2014; Tomback et al. 2014, 2016). Tree island initiation involves the initial establishment of an isolated or *solitary* krummholz conifer followed over time by the establishment of other conifers in its lee. The facilitation provided by an established solitary tree leading to tree island initiation may be important for different life‐history stages for leeward conifers, providing one or all of the following: (1) a safe site and a suitable microsite for seed germination, (2) microsite protection leading to seedling survival, and (3) windward protection for krummholz trees (Smith et al. 2003; Resler et al. 2005; Maher and Germino 2006; Malanson et al. 2007). In general, the survival of first‐year seedlings at treeline is extremely low (Smith et al. 2003), and recruitment may either be at low rates or episodic, coinciding with optimal conditions.

Previous studies show *P. albicaulis* to be the most prevalent solitary tree in many, but not all Rocky Mountain treeline communities (Tomback et al. 2014, 2016). For 10 study areas across the Rocky Mountain distribution of *P. albicaulis*, Tomback et al. (2016) found that proportional abundance of *P. albicaulis* as a solitary tree predicted its proportional abundance as a tree island initiator, but did not predict this strongly for associated conifers. Differences in the prevalence of *P. albicaulis* as an initiator were not latitude‐related, but possibly climate‐related (Resler et al. 2014). For example, in Kootenay National Park, British Columbia, *P. albicaulis* initiated >70% of tree islands, but farther east in Banff National Park at the same latitude, no *P. albicaulis* initiated tree islands (Tomback et al. 2014).

*Pinus albicaulis* depends on *Nucifraga columbiana* (Clark's nutcracker) for seed dispersal (Hutchins and Lanner 1982; Tomback 1982). Nutcrackers often cache seeds near nurse objects (Tomback 1978, 1986), and *P. albicaulis* seedlings tolerate poor soils, drought, and high solar radiation (Arno and Hammerly 1984; Tomback et al. 2001; Maher et al. 2005). These factors may increase *P. albicaulis* dispersal to, and survival at, treeline (Maher et al. 2005; Resler et al. 2005; Resler and Tomback 2008). The survival in general, however, of *P. albicaulis* in subalpine communities and at treeline in the Northern Rocky Mountains is increasingly threatened by white pine blister rust, a frequently fatal disease of five‐needle white pines caused by the non‐native fungal pathogen *Cronartium ribicola* (Tomback and Achuff 2010). Infected small‐diameter trees may die within a few years (Tomback et al. 1995), which may limit the opportunity for tree island initiation, or reduce the survival of tree island components at different life‐history stages. This potentially impacts conifer community structure at treeline.

The frequency of *P. albicaulis* as a tree island initiator may be explained in part by its relative abundance as a solitary tree, but also by the quality of leeward microsite protection provided. In some Northern Rocky Mountain treeline communities, *P. albicaulis* is the most frequent solitary tree, but not a frequent tree island initiator (Tomback et al. 2014, 2016). In some communities where it is infrequent, it has been shown to provide facilitation leading to conifer establishment on harsh sites (Habeck 1969). Using a block design controlling for local topography and climate, Pyatt et al. (2016) found that microsites leeward of both solitary *P. albicaulis* and *Picea engelmannii* (Engelmann spruce) experienced more moderate microclimate than rocks or exposed microsites. Furthermore, Pyatt et al. (2016) found that microsites leeward of *P. albicaulis, P. engelmannii*, and *Abies lasiocarpa* (subalpine fir) had low sky exposure, which reduces solar radiation and increases nighttime temperatures and water availability (Maher et al. 2005). *P. albicaulis* microsites had the lowest percent sky exposure of all three conifers (Pyatt et al. 2016). Thus, the leeward microsite protection provided by *P. albicaulis* may be similar to or exceed that provided by other common treeline conifers, but this bears further study (Pyatt et al. 2016). If *P. albicaulis*, however, is especially stress tolerant and more likely to survive some life‐history stages than associated conifers, this would help explain its prevalence as a tree island initiator.

Working in two treeline communities on the eastern Rocky Mountain Front, we examined whether *P. albicaulis* may facilitate the early life‐history stages of tree island initiation and also provide protection for leeward krummholz trees. We compared the quality of protection provided by microsites leeward of *P. albicaulis*,* P. engelmannii*, rocks, and in exposed (unprotected) microsites by examining germination of sown seeds and cotyledon seedling survival and survival of planted seedlings. In one study area, we experimentally examined whether the death of windward *P. albicaulis* (simulating tree mortality from blister rust) potentially reduced the growth and vigor of the leeward conifer.

Our response variable for the latter assessment was change in shoot length. Conifer shoot length is influenced by factors including growing season duration, temperature, photoperiod, tree vigor, and soil conditions (Kozlowski 1964). In general, conifer shoot lengths and growth rates decline with conditions associated with stressful environments, in particular cold temperatures, short growing seasons, and poor soils (e.g., Schoettle 1990; Ishii et al. 2007; Reinhardt et al. 2011; Körner 2012). We determined whether shoot length declines from the subalpine to treeline, demonstrating general response by trees to increasing stress, as well as response of the leeward conifer to loss of the windward *P. albicaulis* at treeline.

We specifically examined: (1) the relative abundance in our study areas of solitary *P. albicaulis* relative to other treeline conifers; and whether (2) *P. albicaulis* provides a more protective leeward microsite for seed germination and seedling survival than *P. engelmannii* and other common treeline microsites; and (3) death of windward *P. albicaulis* leads to a reduction in shoot length in leeward conifers, which we suggest is a response to increased environmental stress.

## Methods

### Study areas

**Figure 2:**
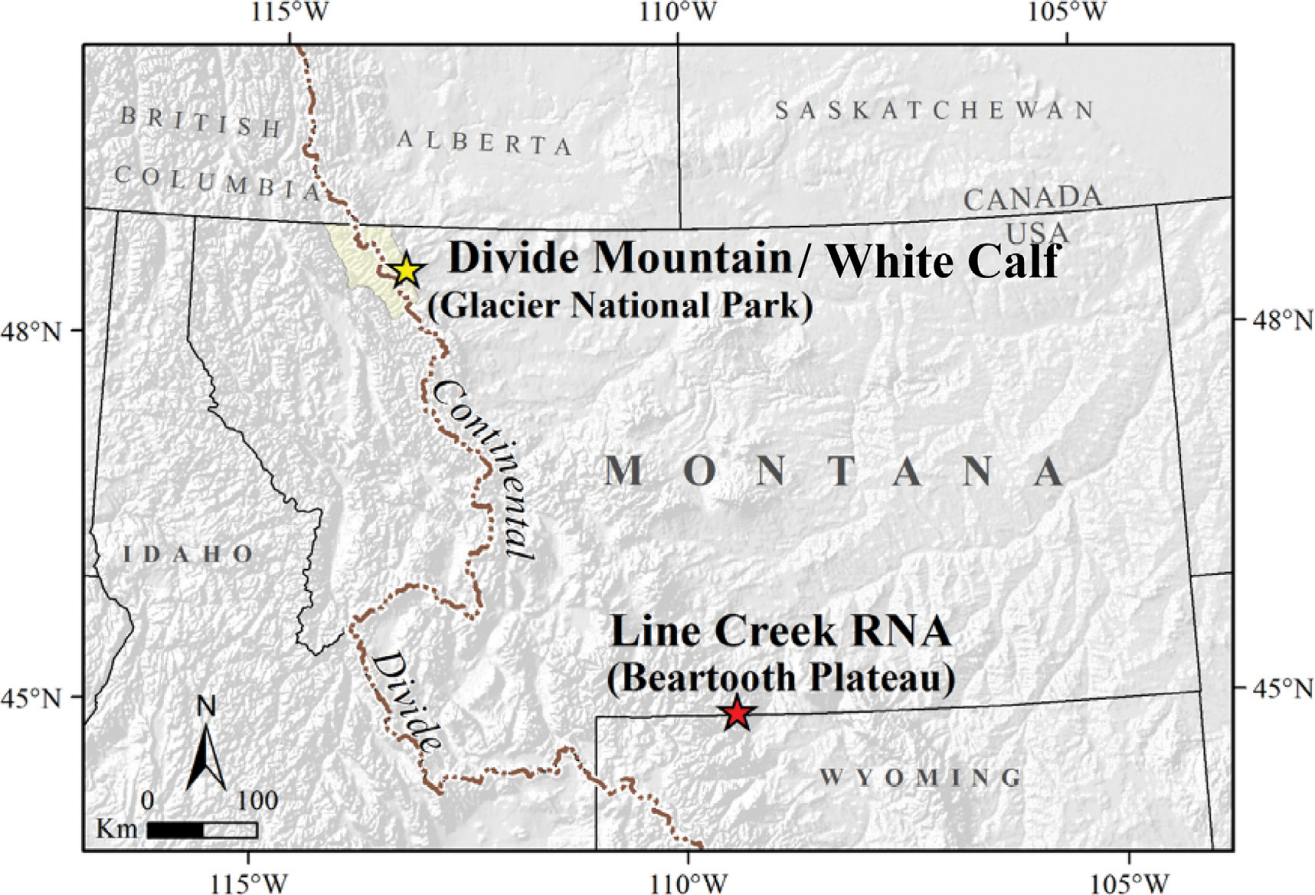
Research study areas include Divide Mountain and adjacent White Calf Mountain, MT, on the eastern slope of Glacier National Park and western Blackfeet Reservation (48°39′25″N, 113°23′45″W); and Line Creek Research Natural Area, Custer National Forest, MT (45°01′47″N, 109°24′09″W). Map modified from Smith‐McKenna et al. (2013).

We conducted research from mid‐July to mid‐September 2010–2013, at two treeline study areas in Montana (Fig. 2). In both study areas, conifers occur as solitary trees, or in tree islands, which are composed of two or more individuals of the same or different species with either contiguous or interwoven canopies. The northern study area included two study sites: Divide Mountain, which straddles the eastern slope of Glacier National Park and the western boundary of the Blackfeet Indian Reservation, at 48°39′25″N lat. and 113°23′45″W lon., and adjacent White Calf Mountain, on the eastern slope of Glacier National Park at 48°38′20″N lat. and 113°24′08″W lon. In both northern study sites, the transition from upper subalpine to treeline communities occurs at about 2100 m; and treeline conifer communities primarily comprise krummholz growth forms of *P. albicaulis*,* A. lasiocarpa*, and *P. engelmannii*. Steep, northeast‐facing slopes characterize the landscape at the study sites in these areas. The bedrock in this region comprises white limestone of the Altyn Formation (Lesica 2002).

The southern study area, Line Creek Research Natural Area (Line Creek), is 530 km to the southeast on the Beartooth Plateau in Custer National Forest (45°01′47″N lat. and 109°24′09″W lon.). There, treeline occurs at 2980 m, with an abrupt transition to krummholz growth forms, dominated by *P. engelmannii* and *P. albicaulis* with fewer *A. lasiocarpa*. Topography at Line Creek is also northeast‐facing, but is less steep than at the northern sites. The soils are shallow, coarse, granitic, and relatively undeveloped (Nimlos et al. 1965).

During our study, microclimates on Divide Mountain were generally warmer and windier and with more precipitation than at Line Creek; however, Line Creek experienced greater daily variation in temperature, less wind in some years, and more frequent freeze‐thaw events than Divide Mountain (Pyatt et al. 2016).

Infection incidences of *P. albicaulis* by *C. ribicola* were determined to be 23.4% for Divide Mountain and 19.2% for Line Creek (Smith‐McKenna et al. 2013).

### Relative abundance and density of solitary trees

We examined relative abundance and density among solitary krummholz *P. albicaulis*,* P. engelmannii*, and *A. lasiocarpa* in observational studies, independent of previous assessments (Resler and Tomback 2008; Smith‐McKenna et al. 2013). In 2012, we used ArcGIS (ESRI 2011) to select 20 random point locations for each study area. At each point, we established a belt transect (50 m × 10 m, 500 m^2^) and recorded the occurrence of all solitary krummholz conifers by species.

### Microsite quality: seed germination and seedling survival

We compared the protective quality of four common treeline leeward microsites (krummholz *P. albicaulis* and *P. engelmannii*, rock, and exposed [open] site) by examining the distribution of seed germination, cotyledon seedling survival, and planted (7‐month‐old) seedling survival among microsite types after 1 year in each study area. In the Divide Mountain study area, no viable *P. engelmannii* seeds were produced in 2010, but we obtained *P. engelmannii* seeds from the appropriate seed transfer zone (USDA Forest Service, Coeur d'Alene Nursery, ID) for sowing in 2011. For the planted seedling study, we collected *A. lasiocarpa* seeds from Divide Mountain in September, 2010; *A. lasiocarpa* was second in abundance to *P. albicaulis* in this area. At Line Creek in 2010, *P. engelmannii* and *A. lasiocarpa* did not produce cones; for the sowing and planting study, we obtained *P. engelmannii* seeds in 2010 from the appropriate seed transfer zone (USDA Forest Service, Bessey Nursery, SD). *P. engelmanni* at Line Creek was second in abundance to *P. albicaulis*. All seedlings were grown by Colorado State Forest Service Nursery for planting in 2011. Seeds for the direct sowing study were chilled for 4 months at 1.7°C before sowing.

In July 2011, in each study area, we identified, marked, and georeferenced 20 replicates each of the four microsite types for the seedling planting and 20 replicates each of the four microsite types for the seed sowing study. We attempted to find *P. albicaulis*,* P. engelmannii*, and rocks of similar heights within a grouping (Table 1). The solitary krummholz conifers at Line Creek were generally twice the height of those at Divide Mountain, reflecting differences in climatic conditions (Pyatt et al. 2016). We determined each leeward microsite position from the branch flagging of nearby krummholz conifers, and either sowed five seeds or planted two seedlings immediately leeward of the microsite nurse object or in the middle of the exposed site. Thus, we sowed *P. engelmannii* seeds in both study areas (seed sowing: 20 replicates × 4 microsite types × 5 seeds per microsite = 400 sown seeds per study area); and we planted *A. lasiocarpa* seedlings at Divide Mountain and *P. engelmannii* seedlings at Line Creek (seedling planting: 20 replicates × 4 microsite types × 2 seedlings per microsite = 160 seedlings). We haphazardly alternated planting and sowing microsites and each microsite type to intermix the experiments and limit them to a subset of the study area with uniform topography and a northeast aspect. We planted each seedling in a 25‐cm‐deep hole to accommodate container root mass and substrate, and marked each seedling with a colored zip tie at the base of the stem. We sowed each seed 0.5‐cm deep in soil. At planting, we provided seedlings with 1.0 L of water, and seeds with 0.5 L. Germination and survival were quantified for all microsite types in July 2012. We revisited cotyledon seedlings (seed germinants) in early September 2012 to quantify summer survival rates.

**Table 1:** Mean and standard deviation (cm) for heights of windward *P. albicaulis* (whitebark), *P. engelmannii* (spruce), and rocks used as leeward microsites for the seedling planting and seed sowing experiment at (a) Divide Mountain and (b) Line Creek. All sample sizes are 20 per microsite for seeds and seedlings at each study area

Site type	Whitebark	Spruce	Rock
(a) Divide Mountain			
Seedlings	19.0 (6.4)	20.9 (6.9)	15.6 (5.8)
Seeds	14.1 (5.0)	13.4 (4.3)	9.9 (2.7)
(b) Line Creek			
Seedlings	44.3 (10.7)	44.8 (12.5)	10.6 (3.8)
Seeds	27.2 (10.8)	28.6 (8.2)	7.2 (2.4)

### Examining the effects of increased stress on shoot lengths

In 2010 at Divide Mountain, we identified isolated solitary krummholz trees, distributed as follows: 17 *P. albicaulis*, 15 *P. engelmannii*, and 15 *A. lasiocarpa*. Each tree was single‐stemmed, nonlayered, and <30 cm in height. The trees were distributed across the study site. These small, solitary trees were uncommon in occurrence and thus selected as encountered. All trees grew under windy, exposed conditions, that is, unsheltered by tree islands or other large “nurse” objects. We georeferenced each tree using a GPS (GeoXT, Trimble GeoExplorer 2008 series, Trimble Navigation Limited, Sunnyvale, CA) and placed tagged nailspikes in the lee of each sampled tree. In September 2011 and 2012, using digital calipers (Mitutoyo 500‐195‐20, Mitutoyo America Corporation, Aurora, IL), we measured to a precision of 0.01 mm the length of five haphazardly selected new branch shoots (total length of the new branch elongation plus extending needles), distributed around the canopy on each tree, after needles were fully extended. Not all trees, however, produced five new shoots every year, so sample sizes varied.

The subalpine forest, with larger stature (upright, i.e., non‐krummholz) trees, occurred at the lower limit of the alpine treeline ecotone. In 2011, five haphazardly selected shoots of each of 10 haphazardly selected conifers of each species at Divide Mountain were measured in September, and at the same time, the krummholz tree shoots were measured. The branch shoots were distributed around the lower canopy and within our reach in order to obtain accurate measurements. In 2012, we increased the sample size to 20 subalpine trees for each species. The measurement procedure was identical to that used for the krummholz trees, except the trees were not marked, so the same trees were not necessarily revisited from year to year.

### Simulating loss of facilitation

In July 2010, we located tree island dyads featuring a windward krummholz *P. albicaulis* sheltering either a leeward *P. engelmannii* (*n* = 40) or *A. lasiocarpa* (*n* = 4) and assigned them to 22 pairs of control and experimental units of the same leeward species. The windward *P. albicaulis* of experimental units were girdled and defoliated. Glacier National Park restricted the girdling of *P. albicaulis* to trees already infected by *C. ribicola*, so the assignment of experimental units was nonrandom. However, the infected trees we selected at treeline were distributed across the northeast‐facing slopes of Divide Mountain and White Calf Mountain. When an experimental (infected) dyad was located, we identified the nearest control (uninfected) dyad. We established 10 experimental–control pairs on White Calf Mountain and 12 pairs on Divide Mountain.

We collected baseline measurements of shoot lengths on the leeward conifer for the five branch shoots nearest to, and directly sheltered by, the windward *P. albicaulis* for all sites in 2010. In some cases, five shoots were not available or there were more than five shoots, in which case the shoots most directly leeward were measured. For sites with extensive canopies leeward of the *P. albicaulis*, we measured only the shoots most directly leeward. After obtaining baseline measurements, we defoliated and girdled the experimental *P. albicaulis*, leaving only a tree skeleton. In 2012, we again selected five branches directly leeward of the windward experimental or control *P. albicaulis* for measurement. In some cases, branches different than those measured in 2010 may have been remeasured, but the criterion was leeward proximity to the windward *P. albicaulis*. In 2013, we removed stem cross sections where possible from the experimental *P. albicaulis* for determining age using standard dendrochronological techniques (Stokes and Smiley 1968).

### Statistical analyses

We used R 2.11.1 (R Core Team 2014) for all analyses. We computed the probability of nonrandom distributions of tree species among the solitary conifers on the 20 transects with multinomial tests; that is, we computed the probability of our observed result if we were to assume that all three species occur with equal probability (*P* = 0.33).

July and September 2012 seed germination counts were compared by microsite type using Fisher's exact probability tests. We used odds ratios to describe the differences in odds of seedling survival at *P. albicaulis* microsite types as compared to the odds of survival at the other three microsite types (e.g., Rita and Komonen 2008). The odds of survival (*S*/(1 − *S*)) at a given microsite type were computed as the proportion of seedlings that survived (*S*) over the proportion of seedlings that died (1 − *S*). The odds ratios for two microsites were computed as the odds of survival at a *P. albicaulis* microsite over the odds of survival at another microsite type. For example, if the odds of survival at *P. albicaulis* microsites were 0.8/0.2 = 4, and the odds of survival at rock microsites were 0.3/0.7 = 0.43, then the odds ratio for survival at *P. albicaulis* microsites as compared with rock microsites would be 4/0.43 = 9.3, indicating that the odds of survival were 9.3 times as great for *P. albicaulis* microsites as for rock microsites. An odds ratio of 1.0 indicates that the odds of survival for seedlings at the two microsites being compared are equal; therefore, any confidence interval around an odds ratio including 1.0 would be evidence for no difference in the odds of survival.

We examined the distribution of shoot lengths for subalpine and krummholz growth forms for *P. albicaulis*,* P. engelmannii*, and *A. lasiocarpa*, by bootstrapping measurements to compensate for multiple shoot measurements from individual trees as follows: We randomly selected 10 trees of each species per year and per zone and randomly selected one shoot per tree. We repeated this process for 1000 iterations and used the 0.025 and 0.975 quantiles of the sampling distribution to identify the low and high endpoints, respectively, of the 95% high density interval (HDI).

To compare shoot lengths measured on subalpine growth forms of each species with their respective counterparts at treeline, we again compensated for nonrandom sampling and multiple samples per tree using a bootstrap analysis. For each comparison, we randomly selected one individual tree from each elevation zone and one shoot per tree. We subtracted the krummholz shoot length (*k*) from the upright shoot length (*u*), and normalized the difference by dividing the quantity by the length of the upright shoot [(*u* − *k*)/*u*]. We repeated the procedure 1000 times for each species and year; we used the 0.025 and 0.975 quantiles of the sampling distribution to identify the low and high endpoints, respectively, of the 95% HDI.

For simulation of loss of facilitation, the sampling of shoot lengths was not balanced because of multiple shoots measured per tree across years. We bootstrapped the differences in shoot lengths for experimental and control dyads as follows: We randomly selected one leeward tree, and for that individual, one shoot measured in 2010 and one in 2012. We then subtracted the length of the 2012 leeward shoot from the 2010 leeward shoot to obtain one measurement of “shoot length difference” for each selected tree. For the bootstrap analysis, we then randomly selected 20 control and 20 experimental tree dyads and calculated the mean shoot length difference for each treatment group. We repeated this 1000 times for each treatment group and again used the 0.025 and 0.975 quantiles of the distribution of bootstrapped data to identify the low and high endpoints, respectively, of the 95% HDI.

## Results

### The relative abundance of solitary krummholz trees

**Figure 3:**
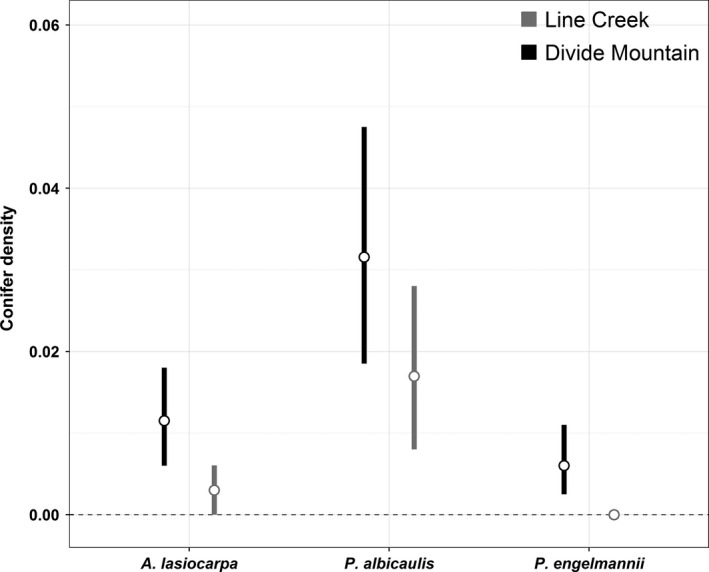
Mean number of solitary krummholz conifers per m^2^ for 20 belt transects each at Divide Mountain and Line Creek. Error bars indicate 95% confidence intervals of the mean.

On Divide Mountain, we counted 487 solitary krummholz conifers across 20 randomly placed transects. Overall species composition comprised 64% *P. albicaulis* (*n* = 312), 23% *A. lasiocarpa* (*n* = 111), and 13% *P. engelmannii* (*n* = 64). On the 20 transects at Line Creek, we found 209 solitary krummholz conifers, including 83% *P. albicaulis* (*n* = 174), 15% *P. engelmannii* (*n* = 32), and 1.4% *A. lasiocarpa* (*n* = 3). Multinomial tests for transects containing one or more solitary conifers indicated a significantly greater abundance of *P. albicaulis* than would be expected under uniform distributions at both Divide Mountain (15/19 transects) and Line Creek (12/15 transects) (Table 2). In both study areas, *P. albicaulis* densities were the highest of the three conifer species, and highest overall at Divide Mountain (Fig. 3).

**Table 2:** Numbers of solitary krummholz conifers by transect for (A) Divide Mountain and (B) Line Creek RNA. For each transect with solitary conifers, the multinomial distribution test probability is computed comparing *P. albicaulis* frequencies to an expected equal distribution for all species

Transect ID	WP	SF	ES	Total	Probability of equal proportions
A. Divide Mountain					
1	18	6	8	32	**0.0037**
2	26	6	6	38	**6.71e‐10**
3	4	0	0	4	**0.012**
4	25	3	6	34	**1.31e‐6**
5	39	10	19	68	**2.073e‐5**
6	14	1	6	21	**0.001**
7	10	0	0	10	**1.53e‐5**
8	15	0	0	15	**5.99e‐8**
9	19	15	1	35	**0.0048**
10	3	16	4	23	0.02
11	3	0	0	3	**0.036**
12	0	0	0	0	n/a
13	2	8	1	11	0.16
14	17	9	0	26	**0.006**
15	22	5	1	28	**8.71e‐7**
16	1	0	0	1	0.33
17	5	8	1	14	0.21
18	10	1	2	13	**0.0013**
19	75	23	9	107	**3.95e‐15**
20	4	0	0	4	**0.012**
Total	312	111	64	487	**15/19**
B. Line Creek					
1	26	0	0	26	**3.03e‐13**
2	3	0	0	3	**0.036**
3	1	0	0	1	0.33
4	11	0	0	11	**5.05e‐6**
5	1	0	0	1	0.33
6	4	0	0	4	**0.012**
7	25	0	0	25	**9.18e‐13**
8	6	1	1	8	**0.02**
9	49	8	0	57	**1.71e‐16**
10	0	0	0	0	n/a
11	12	12	2	26	0.059
12	4	1	0	5	**0.04**
13	12	9	0	21	**0.013**
14	0	0	0	0	n/a
15	0	0	0	0	n/a
16	0	0	0	0	n/a
17	7	0	0	7	**0.00043**
18	8	1	0	9	**0.00085**
19	0	0	0	0	n/a
20	5	0	0	5	**0.0039**
Total	174	32	3	209	**12/15**

WP, *P. albicaulis*; SF, *A. lasiocarpa*; ES, *P. engelmannii*.

Bolded probabilities indicate transects with significantly *higher* frequency of solitary *P. albicaulis*. The total number of transects with higher than expected numbers of solitary *P. albicaulis* is reported at the bottom of the probability column.

### Microsite quality, seed germination, and seedling survival

By 2012, on Divide Mountain, the planted *A. lasiocarpa* seedlings experienced 90% mortality, whereas at Line Creek, overall mortality of *P. engelmannii* seedlings was 63.1%. On Divide Mountain, the surviving seedlings were distributed among microsite types as follows: *P. albicaulis* microsites – 31% (*n* = 5), *P. engelmannii* microsites – 19% (*n* = 3), rock microsites – 12.5% (*n* = 2), and open microsites – 37.5% (*n* = 6). At Line Creek, the surviving seedlings were distributed among the same microsites as follows: *P. albicaulis* microsites – 22% (*n* = 13), *P. engelmannii* microsites – 24% (*n* = 14), rock microsites – 30.5% (*n* = 18), and open microsites – 24% (*n* = 14). The pattern of survival among microsite types was not distinguishable from uniform in either the Divide Mountain or Line Creek locations (exact multinomial test *P* = 0.539 and *P* = 0.824, respectively).

On Divide Mountain, 80 or 20% of 400 *A. lasiocarpa* seeds germinated, with distribution per microsite as follows: *P. albicaulis* – 12, *P. engelmannii* – 17, rock – 32, and open – 19. Higher than expected germination occurred in rock microsites and fewer than expected in *P. albicaulis* microsites (Fisher's exact test, *P* = 0.01). At Line Creek, only 7 (1.8%) of 400 *P. engelmannii* seeds germinated, with no significant difference in germination among the four microsite types (Fisher's exact test, *P* = 0.44).

On Divide Mountain, 42 of 80 cotyledon (new) seedlings survived until September: *P. albicaulis* – 11, *P. engelmannii* – 8, rock – 18, and open – 5; and survival differed among microsite types (Fisher's exact test, *P* = 0.004). Odds of cotyledon seedling survival during summer in *P. albicaulis* microsites were approximately seven times higher than for *P. engelmannii* microsites, 10 times higher than for rock, and 14 times higher than for open microsites (Table 3). Thus, for Divide Mountain, *P. albicaulis* leeward microsites did not increase the odds of seed germination, but did increase the odds of summer survival for cotyledon seedlings.

**Table 3:** Odds ratio analysis of cotyledon seedling summer survival for *P. albicaulis* microsites compared with other microsite types at Divide Mountain. Survival was too low at the Line Creek study area for odds ratio analysis

Microsite comparison	Survival odds ratio	95% LCL	95% UCL
WP/WP	1.00	N/A	N/A
WP/ES	7.14	1.18	43.19
WP/Rock	9.55	1.77	51.44
WP/Exposed	14.00	2.25	87.24

WP, *P. albicaulis*; ES, *P. engelmannii*.

Odds ratios are interpreted as the x‐fold increase in odds of survival at WP sites as compared to odds of survival at other sites. For example, the odds of seedling survival at WP microsites were 14 times that of exposed (open) microsites.

### Shoot length comparisons between subalpine and treeline krummholz conifers

**Figure 4:**
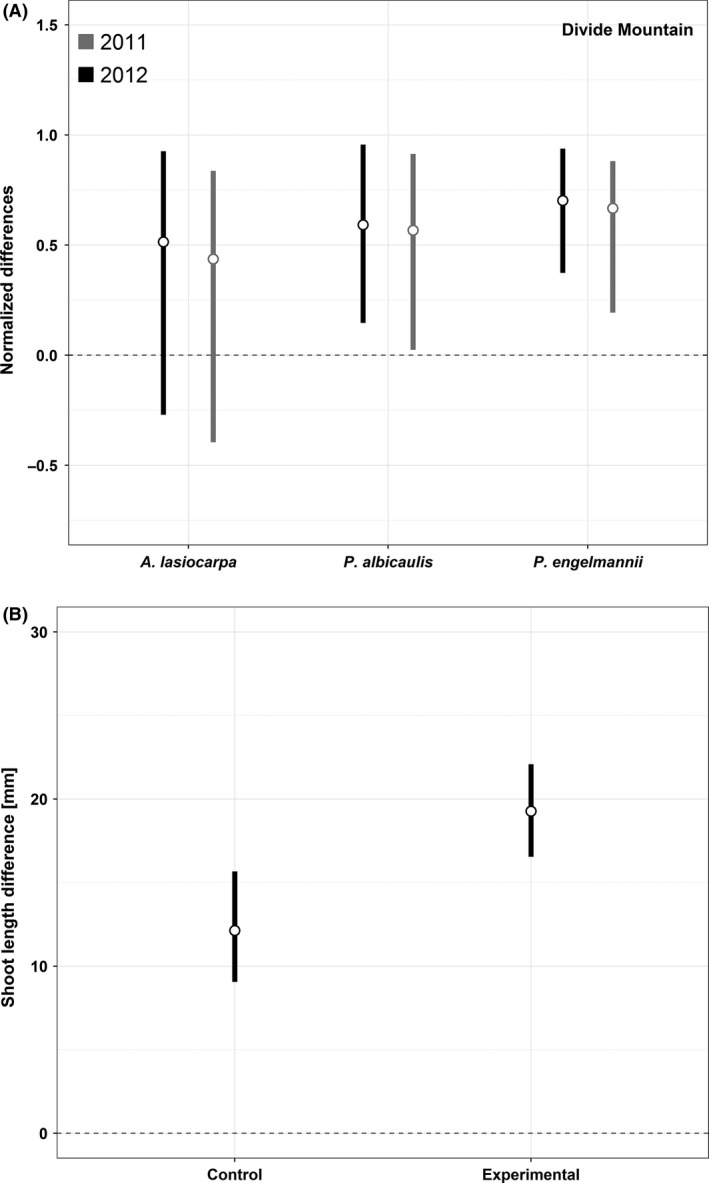
(A) Comparison of bootstrapped shoot lengths between subalpine upright tree growth forms and krummholz *P. albicaulis*,* P. engelmannii*, and *A. lasiocarpa* measured in 2011 and 2012 on Divide Mountain. Mean differences are indicated by the open circles, and lines indicate the extent of the 95% HDIs. Intervals entirely above the 0 line for *P. albicaulis* and *P. engelmannii* indicate that the upright shoots were generally longer than the krummholz shoots. (B) Bootstrapped differences in shoot lengths between initial measurements in 2010 and measurements in 2012 for the conifer leeward of girdled and defoliated (experimental) versus intact (control) *P. albicaulis*. Positive values indicate a shortening of shoot length over time and thus greater differences between 2010 and 2012 measurements. The experiment simulated the loss of facilitation as a result of *P. albicaulis* mortality from *C. ribicola*. HDI, high density interval.

Descriptive statistics for the bootstrapped distribution of shoot lengths from Divide Mountain indicate that krummholz tree growth forms generally had much shorter shoots than subalpine (upright) growth forms in both 2011 and 2012 (Table 4). For *P. albicaulis* and *P. engelmannii* in both years, the normalized bootstrapped differences between shoot lengths for subalpine and krummholz growth forms indicated nonzero differences (Fig. 4A). These results indicate that the length of foliage‐bearing new shoots was shorter for both conifer species at treeline elevations relative to subalpine elevations for this study area in these years.

**Table 4:** Descriptive statistics (mean, minimum, maximum, and lower 0.025 and upper 0.975 quantiles of the 95% HDIs) for bootstrapped shoot lengths (mm) based on measurements in 2011 and 2012 from subalpine zone upright growth forms and krummholz trees from nearby treeline communities on Divide Mountain. The sample sizes for krummholz trees were *P. albicaulis* – 17; *P. engelmannii* – 15; *A, lasiocarpa* – 15. In 2011, we measured shoots on 10 subalpine trees (upright growth forms) of each species and in 2012, 20 trees of each species

Study area	Species	Year	Tree type	Min	Lower	Mean	Upper	Max
Divide	*P. albicaulis*	2011	Upright	39.42	41.35	54.75	68.41	70.89
	*P. engelmannii*			15.86	18.69	33.61	69.44	72.52
	*A. lasiocarpa*			10.27	12.63	26.99	51.94	56.56
	*P. albicaulis*	2012		46.15	47.45	67.05	109.02	124.43
	*P. engelmannii*			16.89	17.15	32.81	71.83	75.29
	*A. lasiocarpa*			8.10	10.78	23.22	43.75	49.35
Divide	*P. albicaulis*	2011	Krummholz	2.72	2.72	22.36	41.53	45.47
	*P. engelmannii*			1.63	2.29	9.08	14.40	14.51
	*A. lasiocarpa*			2.06	2.28	11.29	28.48	29.50
	*P. albicaulis*	2012		6.40	6.40	28.14	61.14	61.14
	*P. engelmannii*			4.06	4.44	10.02	21.86	23.58
	*A. lasiocarpa*			3.96	3.96	11.78	25.78	38.38

### Simulating loss of facilitation

After baseline measurements in 2010, three of 22 (~14%) control *P. albicaulis* were infected by blister rust, died over the course of the study, and were removed from analysis. This observation highlights the rapidity of infection and loss of small‐diameter trees from blister rust. The girdled and defoliated krummholz windward *P. albicaulis* that were sampled ranged in age from 20 to 124 years (*n* = 17, $\overset{¯}{x}$ = 52.9 years, SD = 32.0). Only three trees exceeded 100 years in age. In all cases, the windward *P. albicaulis* was taller or similar in height to the shoots measured on the leeward conifer (Table 5).

**Table 5:** For the experiment simulating the death of *P. albicaulis* from infection by *C. ribicola*, heights (cm) of experimental and control windward *P. albicaulis* (WP) and experimental and control leeward trees (*P. engelmannii* and *A. lasiocarpa*)

Location	Tree type	Mean	SD	Max	Min
Divide	Experimental WP	56.5	23.6	109.0	32.0
	Experimental Leeward	57.6	32.4	138.0	25.5
	Control WP	50.8	23.7	94.0	25.0
	Control Leeward	49.7	32.9	140.0	20.0
White Calf	Experimental WP	82.6	41.4	162.0	29.0
	Experimental Leeward	59.9	20.1	82.0	11.0
	Control WP	69.7	28.4	90.0	36.0
	Control Leeward	59.6	28.4	125.0	26.0

Two years after girdling and defoliation of the experimental windward *P. albicaulis*, shoots of experimental leeward conifers were much shorter than those of control leeward conifers (Table 6). The central 95% of the distributions of bootstrapped shoot length differences (2010 shoot lengths − 2012 shoot lengths) for experimental versus control data did not overlap, indicating that the differences were larger for the experimental sites than for the controls (Fig. 4B). These results suggest that intact, windward *P. albicaulis* offered protection for leeward conifers, and that shoot growth was greater with this protection.

**Table 6:** Descriptive statistics (mean, minimum, maximum, and lower 0.025 and upper 0.975 quantiles of the 95% HDIs) for bootstrapped shoot lengths (mm) measured from the leeward krummholz tree (either *P. engelmannii* or *A. lasiocarpa*) of each experimental (*N* = 22) and control dyad (originally, *N* = 22)

Year	Treatment	Min	Lower	Mean	Upper	Max
2010	Control	12.02	12.56	26.19	51.18	66.02
2012	Control	0.00	0.00	14.01	37.27	42.46
2010	Experimental	9.82	11.04	24.55	45.08	51.50
2012	Experimental	0.00	0.00	5.22	16.73	22.36

## Discussion

### *Pinus albicaulis* relative abundance and stress tolerance

First, we examined the relative abundance of solitary *P. albicaulis* at both Divide Mountain and Line Creek. We found *P. albicaulis* to be the most common solitary conifer species, and thus occurred at the highest density of all conifers, in both study areas. Previous assessments with different sampling designs similarly found *P. albicaulis* to be the most common solitary conifer and the most frequent tree island initiator at both Divide Mountain and Line Creek (Resler and Tomback 2008; Resler et al. 2014; Tomback et al. 2016). Tomback et al. (2016) determined that the proportional abundance of solitary *P. albicaulis* among 10 Rocky Mountain treeline study areas predicted its proportional abundance as a tree island initiator, but the relationship was weaker for both *P. engelmannii* and *A. lasiocarpa*. This suggests that as *P. albicaulis* succumbs to *C. ribicola*, opportunities for tree island initiation may decline.

Divide Mountain and Line Creek have relatively harsh treeline environments. Both study areas are east of the continental divide and exposed to a continental climate, although the climate on Divide Mountain is tempered by maritime influence (Finklin 1986). Pyatt et al. (2016) found that Divide Mountain generally experiences stronger winds than Line Creek, but Line Creek experiences more extreme temperatures and more freeze‐thaw events. The abundance of *P. albicaulis* in both study areas and elsewhere at treeline in the Rocky Mountains may reflect its tolerance of extreme conditions, including poor soils, high winds, and intermittent moisture stress (Arno and Hoff 1990). McCune (1988) classified *P. albicaulis* as a stress‐tolerant pine (sensu Grime 1977).

Stress tolerance in *P. albicaulis* has been substantiated by recent studies examining life‐history and physiological traits. Callaway et al. (2000) determined that *P. albicaulis* at subalpine elevations allocates relatively more biomass to sapwood than to leaves in comparison to *A. lasiocarpa*. Bansal et al. (2011) compared *P. albicaulis* and *P. engelmannii* seedlings grown at treeline and determined that *P. albicaulis* tolerated exposed microsites better; had greater carbon gain, greater carbon use efficiency, and greater water use efficiency in exposed microsites; greater resistance to low‐temperature photoinhibition; and greater soluble sugar concentrations, which may protect against low temperatures. They also determined that young *P. albicaulis* seedlings had lower specific leaf area, which may increase stress tolerance. Thus, the carbon allocation strategies and physiology of *P. albicaulis*, which arise from its ecological niche in the upper subalpine zone characterized as early successional and moderately shade intolerant on productive sites but persistent as a climax species on exposed, windy sites (e.g., Arno and Hoff 1990), are also adaptive for the harsh conditions at many Rocky Mountain treeline sites. The widespread occurrence of *P. albicaulis* at treeline may be due both to effective seed dispersal by Clark's nutcrackers and survival of trees under these harsh conditions.

### Facilitation and early stages in tree island initiation

We compared the leeward microsite protection for planted seedlings, and also the conditions for seed germination and cotyledon seedling survival provided by common microsite types, *P. albicaulis*,* P. engelmannii*, and rocks, as well as exposed microsites in each study area. These experiments examined three life‐history stages and potentially important interactions in tree island initiation, whereby seeds may germinate at higher frequencies, or cotyledon or first‐year seedlings may survive at higher frequencies, if protected by a windward nurse object.

We found that among the surviving planted seedlings, after 1 year there were no differences in their distribution among microsites, but survival rates were generally low. On Divide Mountain, sown seeds germinated best in microsites leeward of rocks. Pyatt et al. (2016), working in the same study areas, found that microsites leeward of rocks had higher soil temperatures in comparison with either exposed microsites or conifer microsites. Higher temperatures in cold environments favor seed germination (e.g., Farmer 1997).

Although sample sizes were small, after 1 year, cotyledon seedlings that germinated from sown seeds had higher odds of surviving the summer in microsites leeward of *P. albicaulis* than in the other microsite types. In general, periodic drought and high UV radiation during summer results in high seedling mortality (Day 1964; Cui and Smith 1991), but *P. albicaulis* microsites appear to moderate these conditions. In the Divide Mountain study area, among the four microsites examined here, *P. albicaulis* leeward microsites had the lowest percentages of sky exposure and slightly higher soil moisture (Pyatt et al. 2016). In general, Pyatt et al. (2016) found that microsites leeward of *P. albicaulis* and *P. engelmannii*, relative to rock and exposed microsites, experienced lower PAR, lower wind speeds, higher minimum and lower variance in soil temperatures, and lower sky exposure. It is unclear why these differences in facilitation quality were not also influential for the distribution of surviving planted seedlings among microsites.

Our results suggest that microsites leeward of *P. albicaulis* may foster survival of new seedlings better than the other microsites examined, although there is discordance with germination. The conditions favoring seed germination, especially higher soil temperatures, may not be favorable for seedling survival in late July and August.

The high seedling mortality rates experienced in our study areas are similar to results obtained by others for first‐year seedling survival at treeline (Malanson et al. 2007). Working with *P. engelmannii* and *A. lasiocarpa*, Smith et al. (2003) noted that first‐year seedling survival was <10%. Germino et al. (2002) found high survival of cotyledon seedlings (80% and higher) during a wet year and 20% or lower in typical years. Natural recruitment may generally occur at low rates or depend on years of optimal conditions (e.g., good seed production, sufficient moisture).

### Simulating loss of facilitation

Our data suggest that shoot length for *P. albicaulis* and *P. engelmannii* is on average shorter at treeline elevations relative to subalpine elevations, a response to increasingly stressful conditions. Shoot length affects the production of new photosynthetic biomass and influences tree architecture (Smith and Brewer 1994; Ishii et al. 2007). Conifer shoot growth is influenced by factors including growing season duration, temperature, photoperiod, tree vigor, and soil conditions (Kozlowski 1964). In general, conifer shoot lengths decline with conditions associated with stressful environments, such as cold temperatures, short growing seasons, and poor soils (e.g., Schoettle and Rochelle 2000; Ishii et al. 2007). Consequently, we used conifer shoot length as a response variable for assessing the effects of lost windward protection (facilitation) for a mature krummholz tree.

Two years after the experimental windward *P. albicaulis* were girdled and defoliated (simulating death from *C. ribicola*), the measured shoots on exposed leeward conifers were significantly shorter than those for control dyads with healthy windward *P. albicaulis*, indicating that the removal of protection increased environmental stress and impacted shoot growth. Windward shelter may be especially important in years with low snowpack and strong winds (Batllori et al. 2009). In addition, loss of the *P. albicaulis* tree island initiator can have negative cascading influences on leeward krummholz trees, especially with respect to wind flow patterns (e.g., Alftine and Malanson 2004; Malanson et al. 2007).

### The impact of *Cronartium ribicola* on Rocky Mountain treeline communities

Our results suggest that widespread mortality of *P. albicaulis* from the introduced, invasive pathogen *C. ribicola* will influence treeline community composition and structure. Between 2010 and 2012, *Cronartium ribicola* infected and killed three of 22 *P. albicaulis* in control dyads, illustrating how rapidly krummholz growth forms may be affected.

Our results confirm that *P. albicaulis* is the most abundant solitary conifer in our study areas east of the continental divide and most likely to initiate tree island development (Resler and Tomback 2008; Smith‐McKenna et al. 2013; Tomback et al. 2016). We also present evidence that *P. albicaulis* provides a protective leeward microsite for cotyledon seedlings; and we demonstrate that it offers windward protection for mature krummholz trees. As *P. albicaulis* declines from the continued spread and intensification of *C. ribicola*, the rate of tree island initiation may also decline. In addition, there may be structural disruption of existing tree islands, not just from loss of the initiating conifer, but also from cascading mortality of windward conifers and loss of *P. albcaulis* within tree islands. In fact, both proportion of stems infected and number of cankers per stem for *P. albicaulis* were higher within tree islands than for solitary trees (Resler and Tomback 2008; Smith‐McKenna et al. 2013).

Losses of subalpine *P. albicaulis* to *C. ribicola* and mountain pine beetle (*Dendroctonus ponderosae*) have reduced seed availability for dispersal to treeline by nutcrackers (McKinney et al. 2009; Barringer et al. 2012). Thus, the composition and structure of treeline conifer communities in some regions may be undergoing rapid change (Tomback and Resler 2007).

Future treeline vegetation dynamics will be highly impacted by global climate change. Warmer temperatures are predicted to shift treeline upward in elevation (Millar et al. 2004; Schrag et al. 2008; Smith et al. 2009), with an estimated elevation gain of 140–700 m (Grace et al. 2002). With fewer *P. albicaulis* at treeline, tree island formation may be delayed or precluded (Tomback and Resler 2007). This outcome has recently been simulated using agent‐based modeling (Smith‐McKenna et al. 2014). Loss of *P. albicaulis* may limit the response of treeline communities to warming temperatures, leading to the perception that treeline is not moving up or moving more slowly than current models for temperature zones would suggest (Tomback and Resler 2007).

## Conflict of Interest

None declared.
